# Tirofiban with sequential dual antiplatelet therapy in mild acute ischemic stroke (TiMIS): protocol for a multicenter, randomized controlled trial

**DOI:** 10.1080/07853890.2026.2644698

**Published:** 2026-03-15

**Authors:** Jiaping Xu, Hao Peng, Yafang Zhu, Fan Xu, Longhai Zhu, Jinping Yang, Tingting Kang, Shuyao Wang, Xianfeng Yu, Junjiang Liu, Penghao Wang, Mingzhi Zhang, Chun-Feng Liu, Yongjun Cao, Jijun Shi

**Affiliations:** a Department of Neurology and Suzhou Clinical Research Center of Neurological Disease, Second Affiliated Hospital of Soochow University, Suzhou, China; b Department of Epidemiology, School of Public Health, MOE Key Laboratory of Geriatric Diseases and Immunology, Suzhou Medical College of Soochow University, Suzhou, China; c Department of Neurology, Taixing Second People’s Hospital, Suzhou Medical College of Soochow University, Taizhou, China; d Department of Encephalopathy, Taicang TCM Hospital, Nanjing University of Chinese Medicine, Suzhou, China; e Department of Neurology, Nuclear Industry 417 Hospital, Xi’an, China; f Department of Neurology, Tongliao People’s Hospital, Tongliao, China

**Keywords:** Tirofiban, dual antiplatelet therapy, mild stroke, early neurological deterioration, antiplatelet therapy, acute ischemic stroke

## Abstract

**Background:**

Patients with mild acute ischemic stroke (AIS) (NIHSS score ≤5) remain at substantial risk for early neurological deterioration (END) and poor functional outcomes despite receiving standard dual antiplatelet therapy (DAPT). Tirofiban, a rapid-onset glycoprotein IIb/IIIa receptor inhibitor, may reduce this risk; however, evidence in patients with mild AIS is limited.

**Objective:**

To determine whether administration of intravenous tirofiban for 48 h followed by DAPT improves the proportion of patients with excellent functional outcomes (modified Rankin Scale [mRS] score 0–1) at 90 days compared with administration of DAPT alone in patients with mild noncardioembolic AIS within 48 h of onset.

**Patients and methods:**

The TiMIS trial is a prospective, multicenter, open-label, blinded-endpoint (PROBE), randomized controlled study. A total of 688 patients aged 18–80 years with noncardioembolic acute mild ischemic stroke within 48 h of symptom onset will be enrolled across 20 centers in China. Participants are randomized 1:1 to intravenous tirofiban for 48 h sequentially followed by DAPT or DAPT alone. The primary outcome is the proportion of patients with excellent functional outcomes at 90 days. Secondary outcomes include END, changes in the NIHSS score, good functional outcome, mRS score shift, incidence of new ischemic stroke and composite cardiovascular events, and safety endpoints (symptomatic intracerebral hemorrhage, all-cause mortality and severe bleeding events).

**Discussion:**

This trial provides crucial evidence on whether early intravenous tirofiban with sequential DAPT offers superior benefits over standard care in patients with mild AIS, potentially guiding future antithrombotic strategies.

**Trial registration:**

ClinicalTrials.gov (NCT07095790).

## KEY MESSAGES

1. This multicenter randomized trial investigates whether early intravenous tirofiban followed by sequential dual antiplatelet therapy improves 90-day functional outcomes in patients with mild acute ischemic stroke compared to dual antiplatelet therapy alone.
2. The study targets the high risk of early neurological deterioration in mild stroke by initiating potent antiplatelet therapy within 48 hours of symptom onset, a critical window for preventing disability.
3. Findings from the TiMIS trial may offer a new therapeutic strategy to enhance recovery and reduce long-term disability in this prevalent yet undertreated patient population.

## Introduction

Mild stroke, which is defined as an ischemic stroke with mild neurological deficits (National Institutes of Health Stroke Scale [NIHSS] score ≤5), accounts for 40%-50% of all ischemic strokes [1]. Despite their initially ‘mild’ presentation, these patients face considerable risks of early neurological deterioration (END), functional disability, and recurrent stroke [2–4]. Notably, a substantial proportion of these patients experience END within 72 h of onset despite receiving standard dual antiplatelet therapy (DAPT), often leading to unfavorable long-term outcomes [5–7]. There is an urgent need to develop better therapeutic strategies for this debilitating disorder worldwide, particularly in China.

Current therapeutic strategies for mild stroke remain challenging. Intravenous thrombolysis has demonstrated no net clinical benefit in recent randomized trials (PRISMS and TEMPO–2), leading to guideline recommendations against its routine use in mild stroke patients with nondisabling deficits [8–10]. Moreover, standard DAPT faces two major challenges: the high prevalence of CYP2C19 loss-of-function alleles in Chinese populations contributes to significant clopidogrel resistance [11,12], and more critically, DAPT fails to prevent END during the crucial 48–72-hour window in a considerable number of patients [13–15]. This limitation highlights a fundamental gap in current antiplatelet strategies. While DAPT inhibits platelet activation *via* two pathways, it may not sufficiently block the final common pathway of platelet aggregation (fibrinogen binding to GP IIb/IIIa) during the hyperacute phase, which tirofiban directly targets.

Tirofiban, a rapid-onset, short-acting glycoprotein IIb/IIIa receptor antagonist, is a promising alternative [16]. Tirofiban may improve microcirculatory perfusion and suppress thrombus propagation by blocking the final common pathway of platelet aggregation, thereby potentially reducing END risk and promoting functional recovery [17–20]. The RESCUE BT2 trial indicated that compared with aspirin, tirofiban could improve functional outcomes in AIS patients without large vessel occlusion (NIHSS score ≥5), although it also highlighted an increased bleeding risk [21]. Recent findings from the TREND trial demonstrated that intravenous tirofiban significantly decreased END incidence without increasing intracranial hemorrhage in mild to moderate stroke patients (NIHSS score 4–20) [20]. Although the trial did not demonstrate improved 90-day functional outcomes in the overall cohort, subgroup analyses suggested that patients with milder strokes—particularly those with atherosclerotic etiology—might derive greater benefit.

Building on this evidence, we hypothesize that in patients with acute, mild, noncardioembolic ischemic stroke within 48 h of onset, compared with DAPT alone, a regimen of early intravenous tirofiban followed by sequential DAPT will increase the proportion of patients who achieve excellent functional outcomes at 90 days, without unacceptable bleeding risk. The TiMIS trial is specifically designed to test this hypothesis and address this critical evidence gap in stroke care.

## Patients and methods

### Study design

Tirofiban in Mild Ischemic Stroke (TiMIS) trial is a multicenter, randomized, open-label, blinded-endpoint, parallel-group study. The protocol was approved by the Ethics Committee of the Second Affiliated Hospital of Soochow University (Approval No. JD-LK2025063) on June 10, 2025, and subsequently by the ethics boards of all the participating centers. The trial was registered at ClinicalTrials.gov (NCT07095790) prior to patient enrollment.

**Figure 1.**
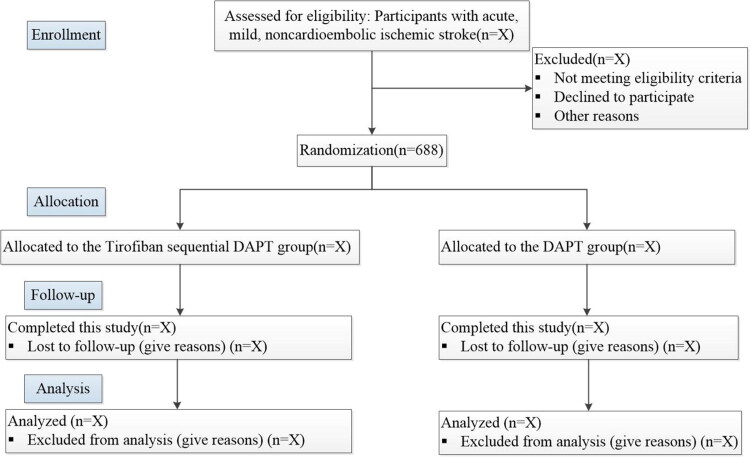
Study flowchart.

From August 2025 to December 2026, a total of 688 patients with acute, mild, noncardioembolic ischemic stroke will be recruited across 20 centers in China. All eligible participants or their legally authorized representatives must provide written informed consent before any study procedures are conducted. The study flowchart is presented in Figure 1, and the schedules of enrollment, interventions, and assessments are summarized in Table 1. The trial will be conducted in full compliance with the principles of the Declaration of Helsinki and the SPIRIT guidelines. Any protocol modifications will be communicated to the ethics committee for approval prior to implementation. The Second Affiliated Hospital of Soochow University is the sponsor of this study. The final findings will be disseminated through publication in a peer-reviewed scientific journal.

**Table 1.** Schedule of patient enrolment, interventions, and outcome assessment.

Procedure/investigation	Baseline	Follow-up 1	Follow-up 2	Follow-up 3	Follow-up 4	Follow-up 5
	Day of randomisation	1^st^ day after randomisation (24h)	2^nd^ day after randomisation (48h)	3^rd^ day after randomisation (72h)	7^th^ day after randomisation or at discharge	90 ± 7^th^ day after randomisation
Baseline data collection						
Inclusion/exclusion criteria check	×					
Acquiring informed consent	×					
Randomisation	×					
Demographic information	×					
Physical examination	×	×	×	×	×	×
Concomitant medication	×	×	×	×	×	×
Demographic information	×					
Physical examination						
Complete blood count^a^	×	×				
Coagulation profile (4 items)	×					
Comprehensive metabolic panel^b^	×				×	
Non-contrast CT/DWI/MR^c^	×					
Efficacy assessment						
NIHSS	×	×	×	×	×	
mRS	×	×	×	×	×	×
New-onset ischemic stroke		×	×	×	×	×
Major adverse cardiovascular events		×	×	×	×	×
Safety observation						
Adverse event/serious adverse event		×	×	×	×	×
All-cause mortality		×	×	×	×	×
Intracerebral hemorrhage		×	×	×	×	×

According to SPIRIT statement of defining standard protocol items for clinical trials.

^a^ Mandatory laboratory tests at screening: A complete blood count (CBC), liver and kidney function tests, and a coagulation profile must be carried out before enrolment. Recommendation: Repeat the CBC within 24 h after initiating tirofiban to monitor for thrombocytopenia.

^b^ Treatment-phase laboratory tests: These must be completed within seven days post-enrolment or at discharge (whichever occurs first) and include: Comprehensive metabolic panel (liver function, lipid profile, fasting glucose, renal function, uric acid, LDH, CK and electrolytes), HbA1c, homocysteine and urinalysis.

^c^ Neuroimaging protocol: A cranial CT scan or MRI scan (preferably an MRI scan with MRA) must be performed within seven days of enrolment or prior to discharge (whichever occurs first) to confirm cerebral infarction. A repeat CT scan must be performed immediately if haemorrhagic transformation is suspected.

### Inclusion and exclusion criteria

The inclusion criteria are as follows: (1) adults aged 18–80 years with noncardioembolic acute ischemic stroke; (2) mild stroke (NIHSS score ≤5); (3) Time from onset to randomization must be ≤48 h; if the time of onset is unknown, time from the last known well to randomization must be ≤48 h; (4) ability to initiate the study medication within 48 h of symptom onset; and (5) written informed consent obtained from the patient or their legal representatives.

The exclusion criteria are as follows: (1) patients who have received or are scheduled to receive intravenous thrombolysis or bridging therapy (subsequent endovascular treatment); (2) a pre‑stroke modified Rankin Scale (mRS) score ≥2; (3) a history of primary intraparenchymal hemorrhage; (4) a history of other intracranial hemorrhage (intraventricular, subarachnoid, epidural, or subdural hemorrhage); (5) untreated or inadequately treated intracranial aneurysm or vascular malformation; (6) major systemic bleeding within 30 days; (7) active bleeding, including laboratory evidence of coagulopathy (platelet count <100 × 10^9^/L, activated partial thromboplastin time >50 s, or international normalized ratio >1.7), or treatment with direct oral anticoagulants within the preceding 48 h; (8) major surgery within 14 days; (9) persistently elevated blood pressure (systolic >180 mmHg or diastolic >110 mmHg) despite treatment; (10) baseline platelet count <100 × 10^9^/L; (11) severe renal dysfunction (glomerular filtration rate <30 mL/min or serum creatinine >220 μmol/L [2.5 mg/dL]); (12) known allergy or contraindication to tirofiban or aspirin; (13) current pregnancy or lactation; (14) any intracranial tumor (except asymptomatic meningiomas ≤1.5 cm in diameter); and (15) any terminal illness with life expectancy <6 months.

### Randomization and blinding

We will apply block randomization to randomly allocate the study participants into the experimental and control groups with a 1:1 ratio. Randomization will be stratified by study center, with the block size undisclosed to maintain allocation concealment. The randomization list is generated by the SAS software PROC PLAN program and kept confidential at the School of Public Health, Suzhou Medical College of Soochow University until eligible study subjects are enrolled. After being fully informed about the protocol of this study and signing the informed consent form, the study subjects will receive a comprehensive assessment to determine if they meet the eligibility criteria. After providing the names of the study subjects enrolled, the physician will have access to the randomization outcome. As an open-label trial, treating physicians and participants are aware of the treatment allocation. However, to ensure unbiased endpoint assessment, we implement a blinded-endpoint (PROBE) design. Specifically, the outcomes at 90 days are performed by independent evaluators. These evaluators are not involved in the participants’ routine clinical care and remain fully blinded to treatment group assignment throughout the study. Furthermore, members of the Clinical Event Committee (CEC) who adjudicate clinical and safety events, as well as the statisticians performing the final analysis, also remain blinded until database lock.

### Intervention

Experimental group (tirofiban sequential DAPT): Participants randomized to the experimental arm will receive intravenous tirofiban immediately after randomization for a total duration of 48 h. This will consist of a loading dose (0.4 µg/kg/min for 30 min) followed by a continuous maintenance infusion (0.1 µg/kg/min for 47.5 h). Beginning at approximately 44 h after tirofiban initiation, oral DAPT with aspirin (100 mg per day) and clopidogrel (75 mg per day) will be started and continued through Day 21, in accordance with previous studies [20,21].

**Figure 2.**
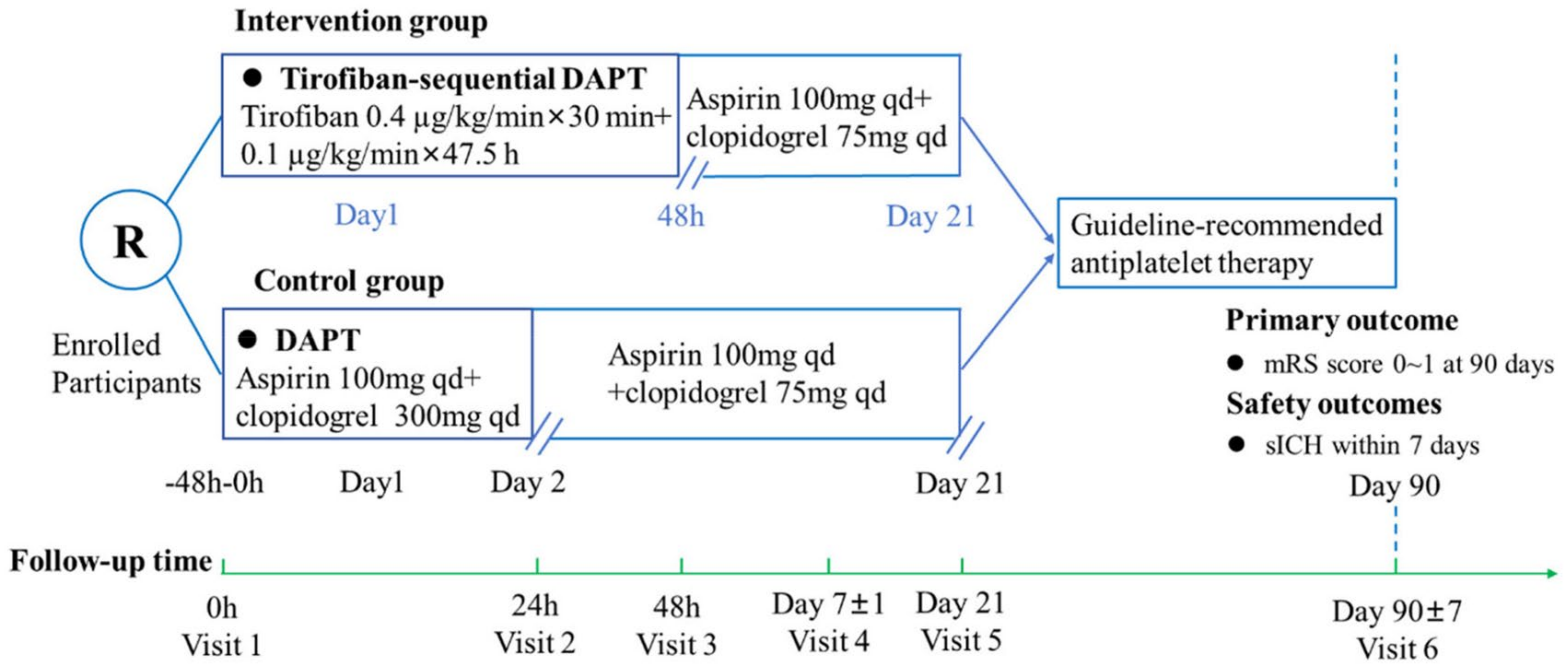
Graphical study design. DAPT, dual antiplatelet therapy.

Control group (DAPT alone): Participants in the control group will receive standard DAPT, initiated promptly after randomization. Participants will receive a loading dose of clopidogrel 300 mg plus aspirin 100 mg on Day 1, followed by maintenance DAPT (aspirin 100 mg daily + clopidogrel 75 mg daily) from Day 2 to Day 21, in accordance with previous studies conducted in Chinese populations (Figure 2) [7,13,14].

For both groups, standard antiplatelet therapy will follow guideline recommendations from Day 22 to Day 90. Any deviation from this should be determined by the treating neurologist according to the established etiology and in compliance with current guidelines.

### Study outcomes

The primary outcome is the proportion of patients with excellent functional outcomes (defined as mRS score 0–1) at 90 days. Secondary efficacy outcomes included END (increase in NIHSS score ≥2 points within 72 h), a change in the NIHSS score from baseline to Day 7, a 90-day good functional outcome (mRS score 0–2), an mRS shift analysis, and the incidence of new ischemic stroke and composite cardiovascular events (including ischemic stroke, hemorrhagic stroke, transient ischemic attack, myocardial infarction, or vascular death).

The primary safety outcome is symptomatic intracranial hemorrhage (sICH) within 7 days, defined according to the ECASS II criteria. Secondary safety outcomes include all-cause mortality and severe bleeding events (GUSTO definition) at 90 days.

END is conservatively defined as an increase of ≥2 points in the total NIHSS score from baseline to 72 h after randomization. NIHSS assessments will be performed by certified on-site raters. Suspected END events are subject to central adjudication by the CEC. The baseline NIHSS score is defined as the initial assessment documented after symptom onset. All efficacy and safety outcomes will be evaluated by assessors who are blinded to treatment allocation.

### Study protocol and data management

The schedule of enrollment, interventions, and assessments is summarized in Table 1. At the baseline visit (Visit 1), trained investigators will screen and enroll eligible patients according to the predefined inclusion and exclusion criteria, incorporating clinical assessment, neuroimaging (MRI), intracranial vascular evaluation, and electrocardiographic findings. Following a comprehensive explanation of the trial protocol—including visit schedules, interventions, and potential risks—written informed consent will be obtained from all participants or their legal representatives.

Eligible subjects will then be randomized *via* a secure web-based central system to either the tirofiban-sequential DAPT group or the control DAPT group. All participants will undergo scheduled follow-up assessments, with a 90-day follow-up conducted in person whenever feasible. All demographic, clinical, laboratory, imaging, and safety data will be prospectively recorded in electronic case report forms (eCRFs) through a password-protected online platform. Local investigators are responsible for verifying the completeness and accuracy of all the entered data before the final submission. The principal investigator and dedicated research staff will perform ongoing, dynamic monitoring of data quality throughout the study period.

### Sample size estimates

The sample size calculation was based on the primary outcome of excellent functional outcome at 90 days. On the basis of previous clinical trials in comparable populations [7,20], the proportion of patients who achieved this outcome was estimated to be 68% in the control group (standard DAPT) and 78% in the tirofiban-sequential DAPT group. To detect an absolute difference of 10% (control 68% vs. intervention 78%) with 80% power and a two-sided alpha of 0.05, 309 participants per group (618 total) are required. Accounting for a 10% dropout rate, the final target sample size is about 688 participants (344 per group). To minimize loss to follow-up, sites will implement structured telephone reminders and flexible visit windows. The study coordinator will track follow-up compliance monthly.

### Data monitoring board

To safeguard participant rights, an independent Data and Safety Monitoring Board (DSMB) comprising academic experts and biostatisticians was established. The DSMB periodically reviews the conduct of the trial, evaluates risk–benefit profiles (paying particular attention to unexpected adverse events) and submits reports containing professional recommendations to the Executive Committee.

### Statistical analysis

The primary outcome is defined as the treatment effect on the proportion of patients achieving an mRS score of 0–1 at 90 days, analyzed according to the intention-to-treat (ITT) population. A chi-square test will be used for the between-group comparison, with results reported as the relative risk and absolute risk difference, each accompanied by a 95% confidence interval. An ordinal analysis of the full mRS distribution (shift analysis) will be performed using ordinal logistic regression, and the treatment effect will be summarized as a common odds ratio. To handle missing data for the primary outcome, multiple imputation will be applied under the missing-at-random assumption. A worst-case scenario sensitivity analysis will also be conducted to assess the robustness of the findings. Efficacy analyses will be primarily based on the ITT population, with supportive sensitivity analyses performed on the per-protocol population. In addition, CYP2C19 genotyping will be conducted as a key exploratory analysis in participants with available genotyping results to assess whether the treatment effect of tirofiban differs according to metabolizer status. Continuous variables will be assessed for normality using the Kolmogorov–Smirnov test. Normally distributed data will be summarized as the mean ± standard deviation and compared using independent samples *t* tests. Nonnormally distributed data will be presented as medians (interquartile ranges) and will be analyzed using the Wilcoxon rank-sum test. Categorical data will be compared using chi-square tests or Fisher’s exact test, as appropriate, and ordinal data will be analyzed using nonparametric methods.

One interim analysis for futility and safety will be conducted by the DSMB after 50% of participants have completed the 90-day follow-up, using O’Brien-Fleming boundaries. For secondary outcomes, binary endpoints will be analyzed in a similar manner; continuous endpoints will be evaluated using linear models; and time-to-event endpoints will be analyzed *via* Cox proportional hazards regression. Sensitivity analyses will be performed to examine the robustness of the findings with respect to missing data and protocol deviations.

All analyses will be conducted using SAS or R software, with a two-sided *p* value < 0.05 considered to indicate statistical significance. This trial is designed as a superiority study, with the primary endpoint being the proportion of patients achieving an excellent functional outcome at 90 days.

### Study organization and funding

The TiMIS trial is an investigator-initiated clinical study chaired by Dr. Jijun Shi. Conducting across 20 clinical centers in China, this prospective, randomized controlled trial follows the SPIRIT guidelines. The steering committee, composed of senior neurologists and trial methodologies, oversees all operational aspects, including protocol implementation, quality control, and endpoint adjudication. An independent DSMB provides ongoing safety oversight and interim analysis review. Clinical endpoints at 90 days are assessed by blinded evaluators to minimize bias.

There was no funding source for this study. Investigational tirofiban is supplied by the sponsoring institution, while standard care medications and procedures are covered through routine medical insurance.

### Current status

As of this submission, patient enrollment for the TiMIS trial is actively in progress. The study commenced on August 25, 2025, and the first participant was recruited on August 29, 2025. The current enrollment is 100 participants. Recruitment will be sustained until the predetermined sample size is complete.

## Discussion

The TiMIS trial addresses a persistent challenge in the management of acute mild noncardiogenic ischemic stroke: how to further improve the rate of excellent functional recovery in an era where standard DAPT has become the cornerstone of treatment. Despite its established benefits, DAPT fails to prevent early neurological deterioration (END) in a clinically significant proportion of patients, particularly those with branch atheromatous disease where END rates approach 40% [7,14,15,22,23]. This limitation reflects the pathophysiology of progressive microthrombosis in penetrating arteries—a process inadequately addressed by oral antiplatelet agents during the critical first 72 h.

Our interventional strategy was designed to target this therapeutic gap. The 48-hour intravenous tirofiban infusion covers the peak END risk period while avoiding prolonged exposure, which might increase bleeding complications. This acute-phase intervention connects a crucial pathophysiological window before a patient transitions to sustained oral DAPT. The subsequent 21-day DAPT course maintains alignment with guideline-recommended secondary prevention [10]. This sequential approach—initiating potent intravenous antiplatelet therapy during the vulnerable phase and then stepping down to oral maintenance—represents a novel therapeutic paradigm for mild stroke management.

Methodological choices in TiMIS reflect practical considerations in contemporary stroke research. The PROBE design balances operational feasibility with endpoint reliability, whereas centralized endpoint adjudication ensures objectivity. Our focus on 90-day functional outcomes rather than END or recurrence rates acknowledges that disability prevention remains the ultimate goal of acute stroke therapy [24]. This patient-centered endpoint captures the net clinical effect of the intervention, which is particularly relevant in mild stroke where traditional efficacy measures may lack sensitivity.

The standardized exclusion of cardioembolic stroke, although potentially limiting individual risk stratification, enhances protocol implementability across multiple centers and ensures timely randomization within the narrow therapeutic window. This pragmatic approach prioritizes generalizability while maintaining scientific rigor.

In summary, the TiMIS trial bridges a critical gap between acute intervention and secondary prevention in mild stroke. The proposed sequential regimen addresses the known limitations of standard DAPT during the first 48 h. These findings will potentially offer clinicians a new therapeutic option, thereby further reducing the risk of disability and improving long-term prognosis for patients with mild stroke, with significant clinical and public health importance.

## Supplementary Material

SPIRIT Checklist.docx

## Data Availability

The full protocol, participant-level dataset, statistical plan, and informed consent materials can be available from the corresponding author on reasonable request after the formal publication of this trial.
